# Seizures and alcohol withdrawal: A literature review

**DOI:** 10.1192/j.eurpsy.2022.2143

**Published:** 2022-09-01

**Authors:** M. Gonçalves, F. Félix, J. Romão, R. André, C. Sereijo, F. Ismail

**Affiliations:** Centro Hospitalar Universitário Lisboa Norte, Psychiatry, Lisboa, Portugal

**Keywords:** Seizures, alcohol withdrawal, delirium tremens

## Abstract

**Introduction:**

Seizures occur in about 3% cases of alcohol withdrawal. They usually appear within 48 hours after abrupt cessation, and are characterized by a reduction in seizure threshold secondary to adaptation to alcohol. More than 50% of individuals will experience a new seizure and in 5% of these cases, progression to a sustained epilepticus status can occur.

**Objectives:**

The aim is to do a review of the literature on alcohol withdrawal and the onset of seizures in individuals with alcohol addiction.

**Methods:**

A literature review was conducted using the PubMed search database.

**Results:**

Alcohol is a central nervous system (CNS) depressant and chronic consumption causes neuroadaptation in order to maintain homeostasis. This adaptation involves the upregulation of excitatory neurotransmitters systems and the downregulation of inhibitory ones. When consumption is abruptly discontinued, the depressive contribution of alcohol to a previously established balance is disrupted, resulting in withdrawal symptoms associated to a generalized CNS’ hyperexcitability state.Critical episodes increase the risk of *delirium tremens*, a fatal condition in 20% of untreated cases. Thus, the treatment and prevention of seizure recurrences is essential: the clinical guidelines of the American Society of Addiction Medicine 2020, offer an action proposal. Pharmacological therapy after seizures is the preferential treatment: intravenous administration of fast-acting benzodiazepines (lorazepam and diazepam) is the first line treatment.

**Conclusions:**

It is essential to monitor signs and symptoms that alert us to the appearance of seizures associated to alcohol withdrawal, effectively treat these cases, prevent recurrences, and provide a quality follow-up for these patients.

**Disclosure:**

No significant relationships.

